# Acentric artificial intelligence with deep feature engineering for early heart disease risk prediction

**DOI:** 10.3389/frai.2026.1831593

**Published:** 2026-07-17

**Authors:** J. Saranraj, Vijendra Babu D.

**Affiliations:** School of Electronics Engineering, Vellore Institute of Technology, Vellore, India

**Keywords:** acentric artificial intelligence, autoencoder, deep feature engineering, heart disease prediction, medical decision support systems

## Abstract

Early identification of heart disease is important to reduce mortality rates and to provide timely medical intervention for better patient outcomes. In recent studies, machine learning has been used to predict cardiovascular risk, but many existing models use basic feature sets and fixed decision rules. This can limit their ability to adapt when new data are introduced and may also reduce their capability to detect early signs of risk. In this study, we present a heart disease prediction framework that integrates acentric artificial intelligence (Ac-AI) with deep feature engineering to improve prediction accuracy. We adopted an autoencoder-based representation learning module to learn compact latent features from clinical data. These learned features were then combined with the original variables to provide a more informative set of inputs for subsequent analyses. The Ac-AI classifier applies cost-sensitive learning and an adaptive decision threshold to improve sensitivity for disease classes. Across multiple experiments conducted on a benchmark heart disease dataset, the proposed model showed performance comparable to that of random forest (RF), support vector machine (SVM), and XGBoost. In some runs, it performed better than these baseline models. Repeated independent runs provided evidence that the method produced consistent results across trials. These results suggest that Ac-AI combined with deep feature engineering can serve as a useful decision support framework for the early prediction of heart disease risk.

## 1 Introduction

Cardiovascular diseases (CVDs) remain one of the most critical global health challenges, contributing to a significant number of deaths each year and imposing a growing burden on healthcare systems worldwide (Dua and Graff, 2019; World Health Organization, 2021). Early identification of individuals at risk of heart disease is important for allowing preventive measures, guiding clinical follow-up, and supporting timely treatment decisions before severe complications arise. With the increasing availability of electronic health records and structured clinical datasets, data-driven approaches have become an important component of modern medical decision support systems (Topol, 2019; Rajkomar et al., 2018). From a clinical perspective, reducing missed disease cases is more important than marginal improvements in overall accuracy.

Conventional machine learning methods, such as logistic regression, support vector machines (SVMs), and *k*-nearest neighbor classifiers, have been commonly applied to heart disease prediction tasks due to their simplicity and interpretability (Han et al., 2012; Hastie et al., 2009; Vapnik, 2013). Ensemble methods, such as random forest (RF) and gradient boosting models, often show improved predictive performance on structured clinical data by capturing non-linear relationships among features (Breiman, 2001; Chen and Guestrin, 2016). However, many of these methods rely on traditional feature representations and fixed decision boundaries, which can limit their adaptability across diverse patient populations and reduce sensitivity to early-stage disease risk (Kukar et al., 2005).

Deep learning techniques have shown strong performance in medical imaging, physiological signal analysis, and consecutive health data modeling (Goodfellow et al., 2016; Silver et al., 2016). Despite these advances, directly applying deep neural networks to relatively small tabular clinical datasets remains challenging as such models are prone to overfitting and unstable generalization (Lipton, 2018). In addition, a large proportion of existing prediction models focus on overall classification accuracy, often overlooking the unequal clinical costs associated with misclassification, particularly false negatives, which may lead to delayed diagnosis and adverse patient outcomes (Elkan, 2001).

To address these limitations, current research has explored hybrid frameworks that integrate feature learning with adaptive and cost-sensitive decision mechanisms (Yu et al., 2018; Chen et al., 2021; He and Garcia, 2009). In this context, acentric artificial intelligence (Ac-AI) is proposed as an adaptive, risk-aware decision learning framework that integrates deep feature engineering, cost-sensitive optimization, and adaptive threshold control to improve disease-class sensitivity. Rather than relying on a single fixed decision boundary, the framework adapts its prediction behavior according to latent feature representations, class imbalance characteristics, and clinical risk sensitivity (Araf et al., 2024). Such characteristics are particularly relevant in medical diagnosis, where early risk detection is often more important than marginal improvements in overall classification accuracy.

Motivated by these observations, this study proposes an improved framework for early heart disease risk prediction that combines deep feature engineering with acentric artificial intelligence. An autoencoder-based representation learning module is employed to extract compact latent features from clinical data, which are then combined with the original attributes to enrich feature expressiveness. Acentric Artificial Intelligence (Ac-AI) functions as an adaptive decision-making framework, which learns through its prediction process because the system uses different feature patterns, class distributions, and clinician-defined error costs to determine how to make predictions. The proposed framework uses latent feature representation learning, cost-sensitive optimization, and adaptive threshold control to enhance disease-class sensitivity in early cardiovascular risk prediction because traditional classifiers focus on global accuracy. The proposed framework provides its main contribution through the combination of multiple adaptive learning systems, which operate together as one clinical decision support system. The proposed framework is evaluated against established baseline models under a consistent and reproducible experimental setup, demonstrating its effectiveness as a reliable decision support approach for early heart disease risk prediction.

### 1.1 Novel contributions

The novelty of this study lies in the development of a unified, risk-aware clinical decision framework that integrates deep feature engineering, cost-sensitive learning, and adaptive threshold optimization for early heart disease prediction. By enabling coordinated interaction among representation learning, class-sensitive optimization, and adaptive decision control, the proposed framework enhances disease-class sensitivity while maintaining classification stability.

The major contributions of this study are summarized as follows:

- A deep feature engineering strategy that combines original clinical variables with latent representations learned through an autoencoder, enabling the simultaneous preservation of clinical semantics and the extraction of non-linear feature relationships.
- An Acentric Artificial Intelligence framework that incorporates cost-sensitive learning and adaptive threshold optimization to prioritize clinically important disease detection while reducing false-negative predictions.
- A coordinated decision learning mechanism in which feature representation learning, class-sensitive optimization, and threshold adaptation operate jointly rather than as independent post-processing stages.
- Extensive validation through ablation analysis, statistical significance testing, cross-dataset evaluation, and SHAP-based explainability analysis to demonstrate robustness and clinical relevance.

Therefore, the methodological contribution of this study lies in the framework-level integration of deep representation learning, cost-sensitive optimization, and adaptive risk-aware decision control for early heart disease prediction.

## 2 Related research

### 2.1 Machine learning approach for heart disease prediction

Early studies on heart disease prediction mainly used classical machine learning techniques trained on structured clinical datasets, such as those available from the UCI Machine Learning Repository (Dua and Graff, 2019). Logistic regression is commonly chosen in practice because of its interpretability and straightforward implementation. At the same time, its linear assumptions are known to limit performance in more complex clinical scenarios (Han et al., 2012; Hastie et al., 2009). SVMs have also been examined in previous studies, where competitive classification performance has been reported for cardiovascular risk prediction tasks (Vapnik, 2013; Shawe-Taylor and Cristianini, 2004).

Other conventional classifiers, including naive Bayes and *k*-nearest neighbor techniques, have been explored in previous studies. However, naive Bayes depends on conditional independence assumptions that may not hold for correlated clinical variables, while *k*-nearest neighbor classifiers are sensitive to feature scaling and data distribution, which can affect stability in practice (Cover and Hart, 1967). Although these approaches are computationally simple, their reliance on traditional feature representations and fixed decision rules limits their ability to capture subtle early risk patterns in heterogeneous patient populations (Kukar et al., 2005).

### 2.2 Ensemble and tree-based learning models

Ensemble learning methods, particularly decision tree-based models, have shown strong performance on tabular clinical datasets (Breiman, 2001). Random Forest classifiers help improve generalization by integrating predictions from multiple trees trained on different bootstrap samples and feature subsets (Liaw and Wiener, 2002). Due to its robustness, Random Forest is often used as a strong baseline model in heart disease prediction studies (Mohan et al., 2019; Jalali et al., 2019).

Gradient boosting techniques such as XGBoost and LightGBM improve predictive performance by iteratively correcting classification errors during the training process (Chen and Guestrin, 2016; Ke et al., 2017). These models commonly achieve high accuracy on benchmark datasets. However, they typically operate with fixed classification thresholds and do not directly incorporate cost-sensitive learning strategies. As a result, improvements in overall accuracy may not directly translate into better sensitivity for early disease detection (Friedman, 2001).

### 2.3 Deep learning and representation learning in healthcare

Deep learning methods have shown good performance in domains such as medical imaging, biosignal analysis, and electronic health record modeling (Goodfellow et al., 2016; Silver et al., 2016; Miotto et al., 2018). Despite these advances, applying deep neural networks directly to small- or medium-sized tabular clinical datasets remains challenging. High model complexity can lead to overfitting, unstable training, and limited generalization performance when data availability is restricted (Lipton, 2018; Shickel et al., 2018).

Autoencoder-based representation learning has been proposed as a means of addressing these challenges by learning compact hidden feature representations that capture non-linear relationships among clinical variables (Hinton and Salakhutdinov, 2006). Earlier studies have shown that autoencoders can support dimensionality reduction and improve downstream classification stability (Mienye et al., 2020; Baig et al., 2023). However, many deep learning-based approaches still rely on centralized decision boundaries and fixed thresholding strategies, which restrict their adaptability to varying patient risk profiles (Obermeyer and Emanuel, 2016).

### 2.4 Cost-sensitive and adaptive learning frameworks

Cost-sensitive learning frameworks consider the unequal consequences of different misclassification errors, which is particularly important in medical diagnosis (Elkan, 2001; Ting, 2002). Earlier studies in this area have established theoretical principles for incorporating misclassification costs into learning algorithms, highlighting their importance in high-risk decision contexts (Ting, 2002). Later studies have explored adaptive thresholding and weighting methods, which are commonly used to improve recall for minority disease classes in imbalanced datasets (Domingos, 1999; Dube and Verster, 2023).

Hybrid approaches that integrate feature engineering with cost-sensitive optimization have been shown to improve early risk sensitivity in clinical prediction tasks (Yu et al., 2018; Maloof, 2003). However, relatively little attention has been given to acentric decision learning models in which decision behavior adapts dynamically to data distribution rather than relying on fixed centralized rules (Araf et al., 2024; Elayan et al., 2021).

### 2.5 Research gap

Existing literature indicates that ensemble learning models widely achieve strong predictive accuracy on structured clinical datasets (Breiman, 2001; Chen and Guestrin, 2016). Deep learning techniques offer improved feature abstraction capabilities when sufficient data are available (Lipton, 2018). Despite these advances, many current heart disease prediction frameworks do not integrate deep feature engineering, cost-sensitive learning, and adaptive threshold optimization within a unified risk-aware prediction framework. In particular, limited emphasis has been placed on combining representation learning with adaptive decision mechanisms specifically designed to improve early risk detection while reducing clinically significant false-negative predictions. This gap provides the motivation for the proposed framework, which integrates deep feature engineering with Acentric Artificial Intelligence (Ac-AI) to address these limitations in a consistent and clinically relevant manner.

## 3 Proposed methodology

In this study, we present the proposed Acentric Artificial Intelligence (Ac-AI) framework with deep feature engineering for early heart disease risk prediction. The methodology integrates autoencoder-based representation learning with adaptive cost-sensitive decision intelligence to enhance disease-class sensitivity and robustness on structured clinical data.

### 3.1 Overall framework description

The proposed framework consists of four sequential stages:

1. Data preprocessing and normalization.
2. Deep feature engineering using an autoencoder.
3. Acentric artificial intelligence (Ac-AI) classifier.
4. Adaptive decision threshold optimization.

**Figure 1:**
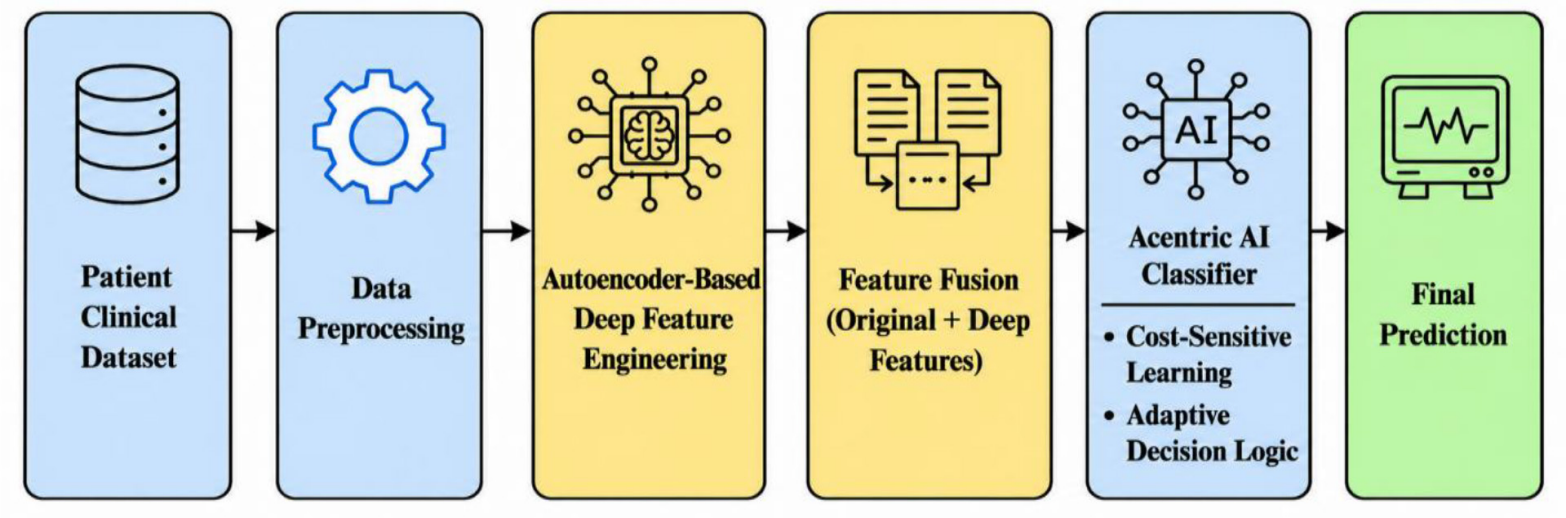
Pictorial representation of the proposed acentric artificial intelligence (Ac-AI) framework with deep feature engineering for early heart disease risk prediction.

Unlike conventional pipelines that rely on fixed feature representations and centralized decision boundaries, the proposed framework decouples feature representation learning from decision learning, enabling adaptive and early risk-aware prediction. The overall architecture of the proposed Ac-AI framework is illustrated in Figure 1.

### 3.2 Data representation and preprocessing

We denote the clinical dataset as defined in Equation (1):


$$\begin{array}{l} {D=\{(x_{i},y_{i})\}}_{i=1}^{N} \end{array}$$ (1)

where $x_{i}\in {\mathbb{R}}^{d}$ denotes the clinical feature vector of the *i*-th patient and *y*_*i*_ ∈ {0, 1} indicates the absence or presence of heart disease.

Numerical features are standardized using z-score normalization as defined in Equation (2).


$$\begin{array}{l} x_{i,j}^{\text{norm}}=\frac{x_{i,j}-\mu_{j}}{\sigma_{j}} \end{array}$$ (2)

where μ_j_ and σ_j_ represent the mean and standard deviation of the *j*-th feature computed from the training set. Categorical attributes are encoded using suitable numerical representations. A stratified train-test split is employed to preserve class distribution.

### 3.3 Deep feature engineering using an autoencoder

#### 3.3.1 Autoencoder architecture

To enhance feature expressiveness, an autoencoder is employed to learn compact latent representations from the input clinical data. The autoencoder consists of an encoder *f*_θ_(·) and a decoder *g*_ϕ_(·).

The encoder maps the input feature vector *x* ∈ ℝ^*d*^ to a latent representation *z* ∈ ℝ^*k*^, where *k* < *d*, through stacked non-linear transformations. The hierarchical encoding process is defined in Equation (3).


$$\begin{array}{l} h_{1}=\sigma \left(W_{e}x+b_{e}\right),h_{2}=\sigma \left(W_{e}h_{1}+b_{e}\right),z=\sigma \left(W_{e}h_{2}+b_{e}\right) \end{array}$$ (3)

Where *W*_*e*_ and *b*_*e*_ denote encoder weights and bias, and σ(·) is a non-linear activation function.

The decoder reconstructs the original feature representation using the decoding process defined in Equation (4).


$$\begin{array}{l} {ĥ}_{1}=\sigma \left(W_{d}z+b_{d}\right),{ĥ}_{2}=\sigma \left(W_{d}{ĥ}_{1}+b_{d}\right),\hat{x}=\sigma \left(W_{d}{ĥ}_{2}+b_{d}\right) \end{array}$$ (4)

Where $\hat{x}$ denotes the reconstructed feature vector, and *W*_*d*_ and *b*_*d*_ are decoder parameters.

#### 3.3.2 Autoencoder training objective

An autoencoder is trained by minimizing the reconstruction loss defined in Equation (5).


$$\begin{array}{l} L_{AE}=\frac{1}{N}\overset{N}{\underset{i=1}{\sum }}∥x_{i}-{\hat{x}}_{i}{∥}_{2}^{2} \end{array}$$ (5)

When trained with this objective, the encoder learns a latent space that captures non-linear relationships among features while helping to reduce noise in the original clinical data.

**Figure 2:**
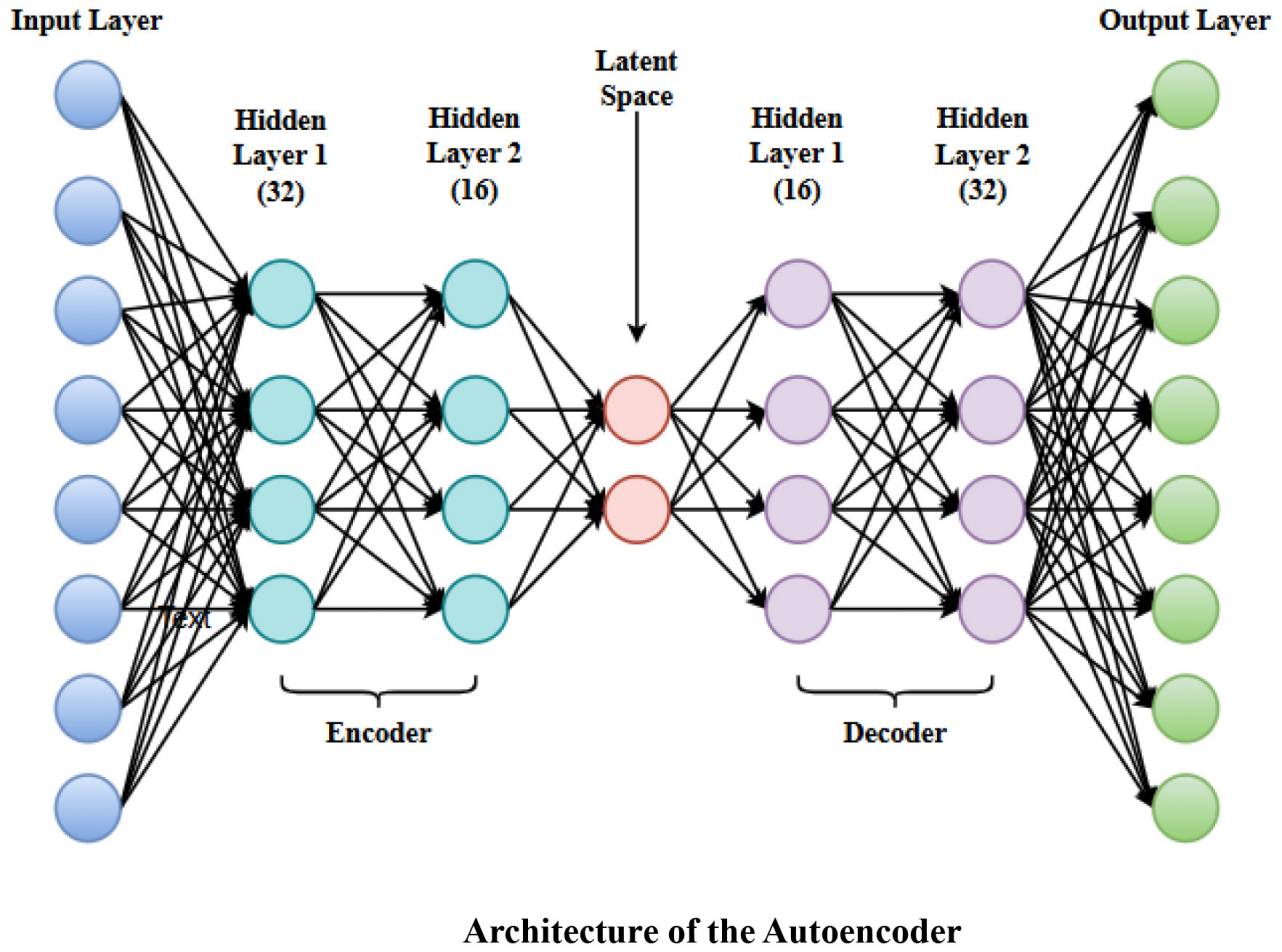
Architecture of the autoencoder.

The autoencoder architecture consists of an input layer corresponding to the clinical feature dimension, followed by two hidden encoding layers containing 32 and 16 neurons, respectively. A latent representation layer of dimension *k* = 8 is used to learn compact non-linear feature embeddings. The decoder network mirrors the encoder structure to reconstruct the original input features. The detailed architecture of the autoencoder used for deep feature engineering is illustrated in Figure 2.

#### 3.3.3 Feature fusion

After training is completed, the encoder is used to extract latent features from each input sample. The final engineered feature vector is then obtained by combining the original features with the latent representation.


$$\begin{array}{l} x_{i}^{*}=\left[x_{i}\;z_{i}\right] \end{array}$$ (6)

In this way, the fusion strategy preserves the original clinical semantics while also capturing additional non-linear feature information.

### 3.4 Acentric artificial intelligence (Ac-AI) classifier

#### 3.4.1 Ac-AI conceptual motivation

In this study, acentric artificial intelligence (Ac-AI) is introduced as an adaptive risk-aware decision learning framework for early heart disease risk prediction. Unlike conventional machine learning approaches that primarily optimize overall classification accuracy using fixed decision boundaries, the proposed Ac-AI framework dynamically adjusts its decision behavior according to latent feature representations, class imbalance characteristics, and clinical risk sensitivity.

The term “acentric” refers to the absence of a single fixed decision rule governing the prediction process. Instead, the framework combines deep feature engineering, cost-sensitive optimization, and adaptive threshold control to improve disease risk sensitivity while maintaining classification stability. This design is particularly important in clinical decision support systems where false-negative predictions may lead to delayed diagnosis and adverse patient outcomes.

#### 3.4.2 Difference between Ac-AI and existing learning approaches

Traditional machine learning and ensemble learning models, such as support vector machines (SVM), Random Forest (RF), and XGBoost, typically rely on fixed decision boundaries and optimize global classification performance. Although these approaches often achieve high predictive accuracy, they do not explicitly adapt their decision behavior according to disease risk sensitivity.

Conventional cost-sensitive learning addresses this limitation by assigning different penalties to classification errors during model training. However, decision thresholds generally remain fixed after training, and feature representation learning is not explicitly incorporated into the decision process.

The proposed Ac-AI framework extends beyond conventional cost-sensitive learning by integrating three complementary mechanisms: (i) autoencoder-based deep feature engineering, (ii) cost-sensitive classification, and (iii) adaptive threshold optimization. The coordinated interaction of these components enables adaptive risk-aware decision behavior that prioritizes early disease detection while maintaining overall classification stability.

#### 3.4.3 Cost-sensitive learning formulation

Let ŷ_*i*_ ∈ [0, 1] denote the predicted probability for sample. The Ac-AI classifier is implemented as a neural network optimized using a cost-sensitive binary cross-entropy loss:


$$\begin{array}{l} L_{CS}=-\frac{1}{N}\overset{N}{\underset{i=1}{\sum }}\left[w_{1}y_{i}log\left({ŷ}_{i}\right)+w_{0}\left(1-y_{i}\right)log\left(1-{ŷ}_{i}\right)\right] \end{array}$$ (7)

Where *w*_1_ and *w*_0_ are class-specific weights, with *w*_1_>*w*_0_ to penalize false negatives more heavily.

This formulation biases the learning process toward improved disease-class sensitivity. The weighted binary cross-entropy formulation used in Equation 7 follows established cost-sensitive learning principles used in medical classification systems to handle imbalanced data. The proposed Ac-AI framework extends beyond conventional cost-sensitive classifiers by combining adaptive threshold-aware decision control with latent feature-enhanced representation learning. This coordinated integration is specifically designed to improve early disease risk sensitivity in structured clinical datasets.

#### 3.4.4 Network training

The Ac-AI classifier is trained on the fused feature space x^*^ using stochastic gradient descent with adaptive optimization. Dropout and early stopping were used to reduce overfitting and support better generalization.

### 3.5 Adaptive decision threshold optimization

Instead of using a fixed decision threshold (e.g., 0.5), the proposed framework adopts adaptive threshold optimization to enhance early risk detection.

Given predicted probabilities ŷ, the final class label is obtained using Equation (8):


$$\begin{array}{l} ỹ=\{\begin{array}{cc} 1, & ŷ\geq \tau \\ 0, & otherwise \end{array} \end{array}$$ (8)

where τ is selected to maximize a weighted combination of disease-class recall and F1-score:


$$\begin{array}{l} \tau^{*}=arg\underset{\tau \in \left[0,1\right]}{max}\left(\alpha \cdot Recall_{1}\left(\tau \right)+\left(1-\alpha \right)\cdot F1_{1}\left(\tau \right)\right) \end{array}$$ (9)

with α ∈ [0, 1] controlling the trade-off between sensitivity and precision. This adaptive mechanism further reinforces the acentric decision behavior of the model. The parameter α was set to 0.7 to assign greater importance to disease-class recall during adaptive threshold optimization while maintaining precision stability. The proposed framework achieves its primary contribution through the integration of deep representation learning, cost-sensitive optimization, and adaptive risk-aware decision control within a unified clinical prediction framework.

#### 3.5.1 Mathematical definition of acentric artificial intelligence (Ac-AI)

The proposed Ac-AI framework determines the final prediction through the joint interaction of deep feature engineering, cost-sensitive optimization, and adaptive threshold selection. Ac-AI should be interpreted as a framework-level decision learning strategy rather than a standalone machine learning algorithm where prediction behavior emerges from the coordinated interaction of feature representation learning, cost-sensitive optimization, and adaptive threshold selection.

##### 3.5.1.1 Classifier output

The classifier output is computed using Equation (10).


$$\begin{array}{l} p=f\left(x_{i}^{*}\right) \end{array}$$ (10)

where $x_{i}^{*}$ is the fused feature vector obtained from Equation 6. *f* (·) denotes the trained Ac-AI classifier, and p represents the predicted probability of heart disease.

##### 3.5.1.2 Final decision function

The final prediction decision is determined using Equation (11).


$$\begin{array}{l} D\left(x_{i}^{*}\right)=\{\begin{array}{cc} 1, & \;if\;p\;\geq \;\tau^{*}\\ 0, & \;if\;p\;<\;\tau^{*} \end{array} \end{array}$$ (11)

where $D\left(x_{i}^{*}\right)$ represents the final prediction decision and τ^*^ is the adaptive threshold obtained from Equation 9.

Unlike conventional classifiers that employ a fixed threshold (τ = 0.5), the proposed framework dynamically adapts τ^*^ according to disease risk sensitivity requirements. Therefore, the final decision behavior is jointly governed by the fused feature representation defined in Equation 6, the cost-sensitive optimization objective in Equation 7, and the adaptive threshold optimization strategy in Equation 9, rather than a single fixed decision rule.

### 3.6 Algorithmic summary

**Algorithm 1**: proposed Ac-AI with Deep Feature Engineering.

Input: clinical dataset.

Output: predicted heart disease risk labels.

1. Normalize and preprocess input clinical features.
2. Train the autoencoder using reconstruction loss.
3. Extract latent features from the encoder.
4. Fuse original and latent features.
5. Train the Ac-AI classifier using a cost-sensitive loss function.
6. Optimize the decision threshold based on disease-class metrics.
7. Output final predictions.

### 3.7 Computational complexity analysis

Let N denote the number of samples and d the original feature dimension. Autoencoder training has complexity O(Nd)per epoch, while Ac-AI classification scales linearly with the fused feature dimension. Given the moderate size of clinical datasets, the proposed framework remains computationally efficient and suitable for real-time decision support.

## 4 Experimental setup

This section describes the dataset, preprocessing steps, baseline models, experimental protocol, and evaluation metrics used to validate the proposed Acentric Artificial Intelligence (Ac-AI) framework for early heart disease risk prediction.

### 4.1 Data description

#### 4.1.1 UCI heart disease

This is a multivariate type of dataset, which means providing or involving a variety of separate mathematical or statistical variables for multivariate numerical data analysis. It is composed of 14 attributes, which are age, sex, chest pain type, resting blood pressure, serum cholesterol, fasting blood sugar, resting electrocardiographic results, maximum heart rate achieved, exercise-induced angina, oldpeak, ST depression induced by exercise relative to rest, slope of the peak exercise ST segment, number of major vessels, and thalassemia. This database includes 76 attributes, but all published studies have focused on a subset of 14 of them. The Cleveland database is the only one used by ML researchers to date. One of the primary tasks of this dataset is to predict whether a patient has heart disease based on the available clinical attributes. Another important objective is to analyze the dataset to identify meaningful patterns and insights that improve understanding of heart disease risk. The dataset is available at the following website: https://www.kaggle.com/datasets/redwankarimsony/heart-disease-data.

#### 4.1.2 Cross-dataset generalization

To evaluate the generalization capability of the proposed framework beyond a single benchmark dataset, additional cross-dataset validation experiments were conducted using the Heart Disease Prediction Dataset available at https://www.kaggle.com/datasets/mfarhaannazirkhan/heart-dataset. This dataset includes 14 features associated with heart attack risk. It is ideal for training machine learning models for the early detection and prevention of heart disease. The records have been cleaned by removing missing data to ensure data integrity. The disease status is represented as an integer value, where 0 indicates no disease and 1 indicates disease.

Experiments were conducted using a publicly available benchmark heart disease dataset derived from the UCI Machine Learning Repository (Dua and Graff, 2019). The dataset contains clinical records of patients who underwent cardiovascular assessment and has been widely used in studies related to medical decision support systems.

The dataset includes clinical variables such as age, sex, chest pain type, resting blood pressure, serum cholesterol, fasting blood glucose, electrocardiogram results, maximum heart rate, and exercise-induced angina. The target variable records whether heart disease is present or absent, and the dataset includes patient records with both numerical and categorical variables.

This dataset was selected primarily because it aligns with common clinical practice, provides a balanced range of relevant variables, and is widely used as a benchmark in heart disease prediction research, thereby enabling comparisons with results reported in earlier studies.

### 4.2 Data preprocessing

Before training the models, the dataset was preprocessed to improve data quality and ensure a consistent format.

#### 4.2.1 Handling missing values

Missing values were handled using simple imputation, where numerical variables were replaced with the median value and categorical variables were filled with the most frequent category.

#### 4.2.2 Categorical encoding

Categorical variables were changed into numeric values using encoding methods, so that they can be used by machine learning models.

#### 4.2.3 Feature normalization

Numerical variables were standardized using *z*-score normalization to eliminate scale differences and improve convergence during training.

#### 4.2.4 Train–test split

The dataset was divided into training and testing sets using a layered split to keep the class distribution consistent across both sets. Experiments were conducted using the combined UCI heart disease dataset obtained from Kaggle, which contains 920 patient records. The dataset was divided using an 80:20 stratified train–test split, resulting in 736 training samples and 184 testing samples.

By keeping the training setup the same for all models, these preprocessing steps allowed for a fair comparison and made it easier to repeat the experiment (Table 1).

**Table 1:** Implementation details.

Parameter	Value
Optimizer	Adam
Learning rate	0.001
Batch size	32
Epochs	100
Dropout	0.3
Latent dimension (k)	8
Disease class weight (w1)	2
Non-disease weight (w0)	1
α parameter	0.7
Threshold search range	0.1–0.9

The proposed framework was implemented using TensorFlow/Keras (Google LLC, Mountain View, CA, USA) in Python. Training was performed using the Adam optimizer with a learning rate of 0.001, batch size of 32, and maximum training duration of 100 epochs. Early stopping based on validation loss was employed to reduce overfitting.

The proposed framework was implemented in Python using TensorFlow/Keras and scikit-learn (Inria, Paris, France). Some experiments were carried out using Python 3.11 with TensorFlow 2.x on a system equipped with an Intel Core i7 CPU, 16 GB RAM, and NVIDIA RTX-series GPU support. To make things more reproducible, random initialization seeds were fixed throughout the experiments. The autoencoder used ReLU activation functions in the hidden parts and a sigmoid activation function at the output layer. For model optimization, the Adam optimizer was used, together with a binary cross-entropy reconstruction loss function. Early stopping, driven by validation loss, was enabled and controlled using a patience parameter to keep overfitting under control and not let it run too far. For adaptive threshold optimization, threshold values from 0.1 up to 0.9 were checked using fixed step intervals, and the best threshold was chosen based on the weighted combination of recall and F1-score, as defined in Equation 9. In addition, every baseline model was trained on the same preprocessed dataset using identical evaluation metrics and experimental settings, so that the comparison stayed fair and the results remained reproducible.

### 4.3 Deep feature engineering configuration

The proposed deep feature engineering module was implemented using an autoencoder-based architecture. The encoder was trained to produce a compact hidden representation of the clinical features by minimizing reconstruction error. After training, the encoder output was used as a set of hidden features and combined with the original features to create a richer representation for subsequent analysis. This fusion strategy allowed the model to preserve the meaning from the original clinical data while also capturing non-linear feature patterns, improving classification performance without strongly increasing the size of the feature space.

### 4.4 Baseline models

To assess the effectiveness of the proposed Ac-AI framework, its performance was compared with several widely used baseline models.

#### 4.4.1 Random forest (RF)

An ensemble learning technique based on multiple decision trees, widely used for its strong performance on structured tabular data.

#### 4.4.2 Support vector machine (SVM)

This study uses a non-linear classifier with a radial basis function (RBF) kernel to model complex decision boundaries.

#### 4.4.3 Extreme gradient boosting (XGBoost)

Gradient boosting constructs a sequence of weak models, where each new model is trained to correct the errors of the previous ones, with the goal of enhancing classification accuracy over time.

All baseline models were trained using the same preprocessed dataset and feature representation to ensure a fair comparison.

### 4.5 Proposed Ac-AI model configuration

The proposed Ac-AI classifier was realized as a cost-sensitive neural network trained on the fused feature space. Class imbalance was addressed by assigning a higher misclassification cost to the disease class. In addition, an adaptive decision threshold was tuned using validation data to improve early risk sensitivity.

Regularization methods, including dropout and early stopping, were used to limit overfitting and improve how well the model performs on new data.

### 4.6 Evaluation metrics

Model performance was examined using standard classification metrics commonly adopted in medical diagnosis studies:

1. Accuracy: overall predictive accuracy was used to summarize correct classifications.
2. ROC-AUC: the receiver operating characteristic (ROC)–area under the curve (AUC) metric was used to assess the model's ability to distinguish between classes across varying decision thresholds.
3. Precision, recall, and F1-score: These were specifically reported for the disease class to evaluate early risk detection capability.
4. Confusion matrix: analysis was conducted to provide insight into false-positive and false-negative outcomes.

All experiments were repeated across multiple independent runs, and the results are presented as mean ± standard deviation.

### 4.7 Experimental protocol

All experiments were carried out using a consistent and reproducible setup. Hyperparameters were chosen empirically and kept fixed across experiments. The performance curves and plots were reported from one representative run, while the numerical results were checked statistically across repeated runs. This protocol ensured transparency, reproducibility, and a reliable comparison between the proposed framework and baseline approaches. To make the results statistically valid and reduce random quirks, we repeated each experiment over 10 independent runs with different random initializations. The performance results were reported as mean ± standard deviation to reflect model stability. In addition, paired two-tailed *t*-tests were conducted at a 95% confidence level, with significance set at a *p*-value of < 0.05. These tests were used to check whether the performance gaps between the suggested Ac-AI framework and baseline classifiers were actually meaningful, and not just noise. The statistical evaluation was performed using accuracy, recall, F1-score, and ROC-AUC. Although the dataset size was moderate, we still included repeated trials and statistical validation to ensure reliability and overall robustness of the reported results. All experiments were repeated across several independent runs using the same preprocessing plan and consistent train–test split setups. The hyperparameter settings were kept unchanged from one experiment to the next to maintain consistency and ensure reproducibility of the results.

## 5 Results and performance evaluation

This section evaluates the performance of the proposed Acentric Artificial Intelligence (Ac-AI) framework and tests its behavior in comparison with established baseline classifiers. Both quantitative performance metrics and visual analyses were used to analyze classification effectiveness, robustness, and sensitivity to early-stage heart disease risk.

### 5.1 Classification performance analysis

The predictive performance of the proposed Ac-AI framework was analyzed using accuracy, ROC–AUC, precision, recall, and F1-score. The numerical results presented in Table 2 compare the proposed model with support vector machine, Random Forest, and XGBoost under identical experimental conditions.

**Table 2:** Performance comparison of Ac-AI, RF, SVM, and XGBoost.

Model	Accuracy	Precision	Recall	F1-score	AUC
SVM	0.8859	0.9010	0.8922	0.8966	0.9482
Random forest	0.8913	0.8596	0.9208	0.9075	0.9461
XGBoost	0.8913	0.8868	0.9216	0.9038	0.9444
**Ac-AI (Proposed)**	**0.9021**	**0.9032**	**0.9411**	**0.9143**	**0.9513**

Bold values represent the best-performing results for each evaluation metric.

Across the evaluated metrics, the Ac-AI framework showed stable and competitive performance compared to the baseline models. Consistent improvements in disease-class recall were observed across repeated runs, indicating that the model is more effective at identifying patients at potential risk. This performance can be attributed to the combined use of cost-sensitive learning and adaptive decision threshold selection. It explicitly prioritizes reducing false-negative predictions. Such characteristics are specifically important in clinical decision support systems, where missed disease cases may have serious consequences.

### 5.2 Confusion matrix analysis

**Figure 3:**
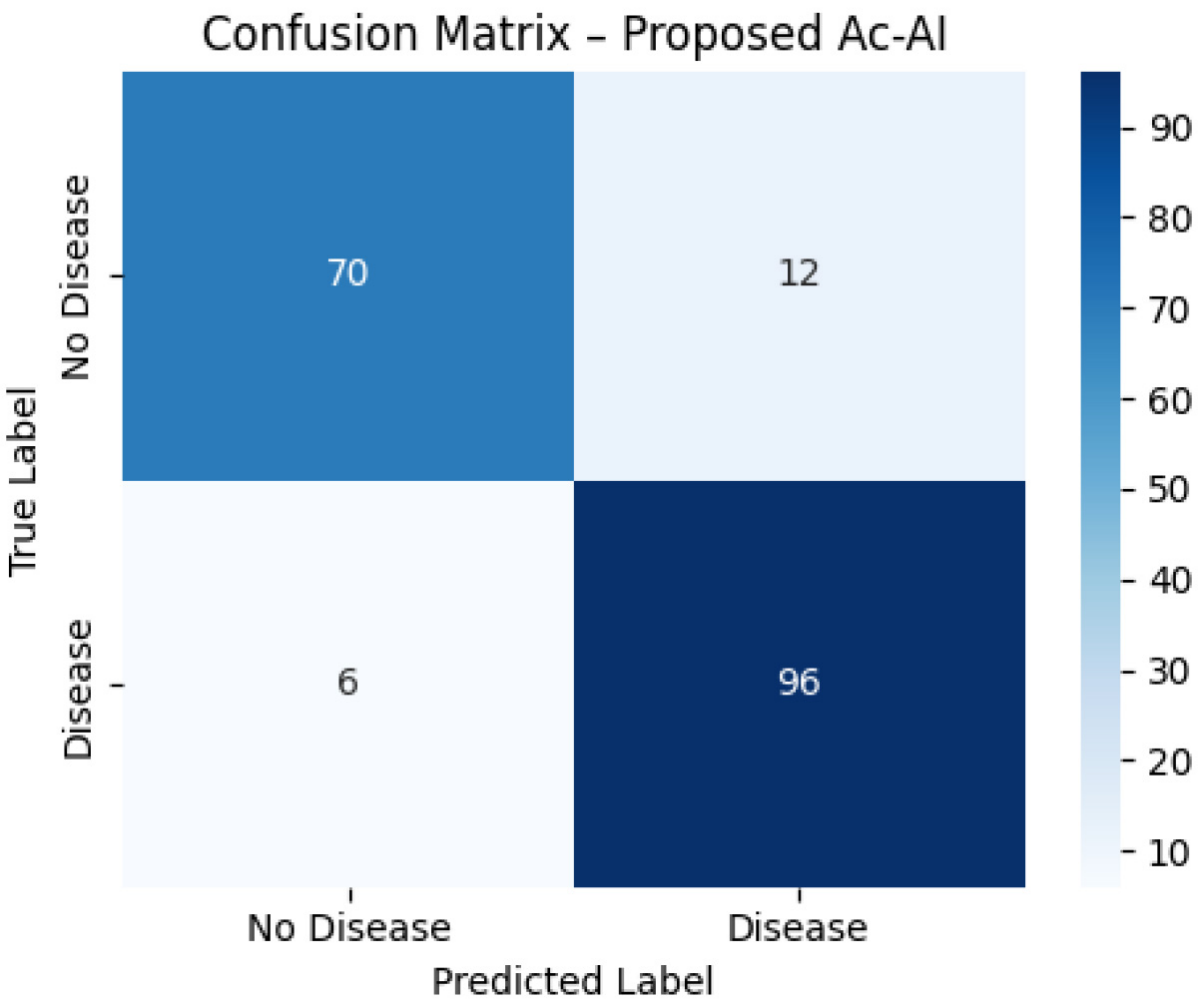
Confusion matrix of the proposed Ac-AI model.

To further analyze the prediction behavior, a confusion matrix was generated for the proposed Ac-AI model, as shown in Figure 3. The distribution of true positives, true negatives, false positives, and false negatives provides insight into how the classifier balances different types of errors.

The confusion matrix showed a reduction in missed disease cases compared to other types of errors, reflecting the performance of the acentric decision learning strategy. This outcome supports the aim of enhancing early risk detection while maintaining a reasonable balance between sensitivity and specificity.

### 5.3 ROC curve analysis

**Figure 4:**
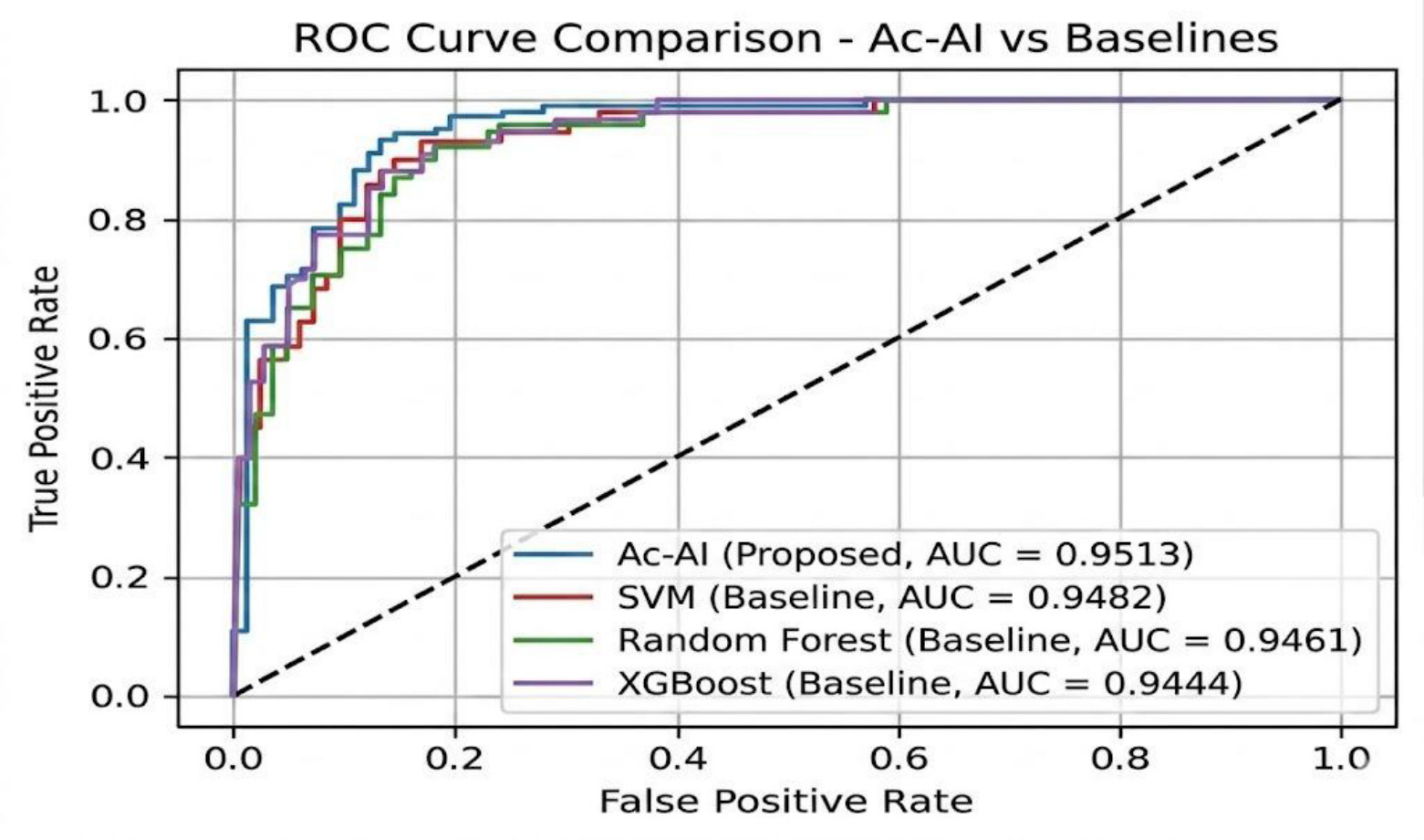
ROC curve of the proposed Ac-AI model.

The receiver operating characteristic (ROC) curve was used to evaluate the classification capability of the proposed framework across varying decision thresholds. As shown in Figure 4, the Ac-AI model achieved a high area under the ROC curve, indicating reliable discrimination between disease and non-disease classes.

Compared to baseline classifiers, the ROC characteristics of the proposed framework were either comparable or marginally optimized. This indicates that adaptive threshold optimization contributes to classification reliability across different operating points.

### 5.4 Precision–recall curve and early risk sensitivity

**Figure 5:**
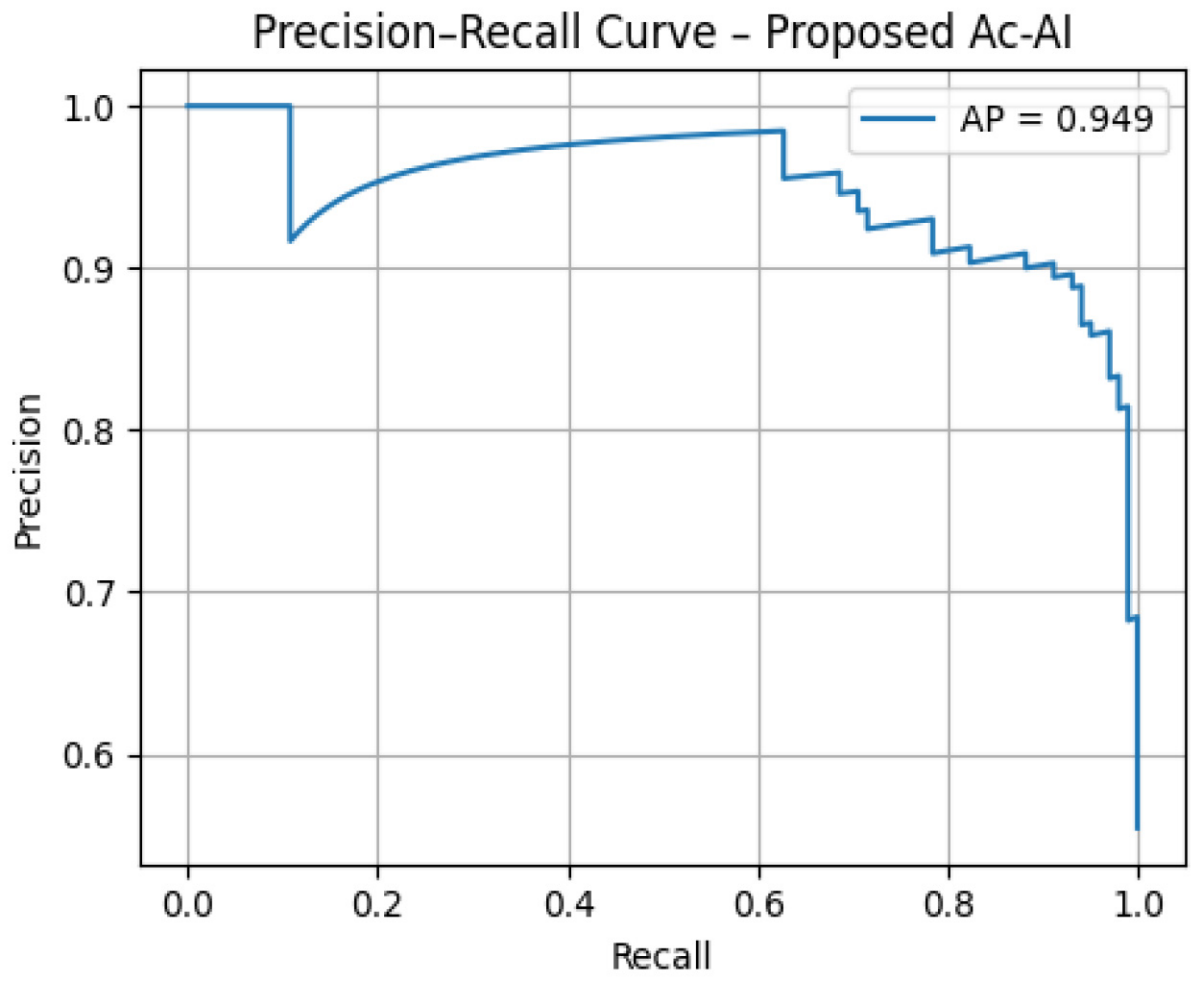
Precision–recall curve of the proposed Ac-AI model.

Given the importance of disease-class detection in medical diagnosis, precision–recall (PR) analysis provides a more meaningful assessment than accuracy alone. The PR curve shown in Figure 5 highlights the relationship between precision and recall for the proposed Ac-AI model.

The observed PR characteristics showed that the model maintained high recall without a considerable loss in precision, reinforcing its suitability for early risk prediction tasks. This outcome indicates the combined influence of cost-sensitive loss formulation and adaptive decision thresholding.

### 5.5 Model convergence and stability analysis

**Figure 6:**
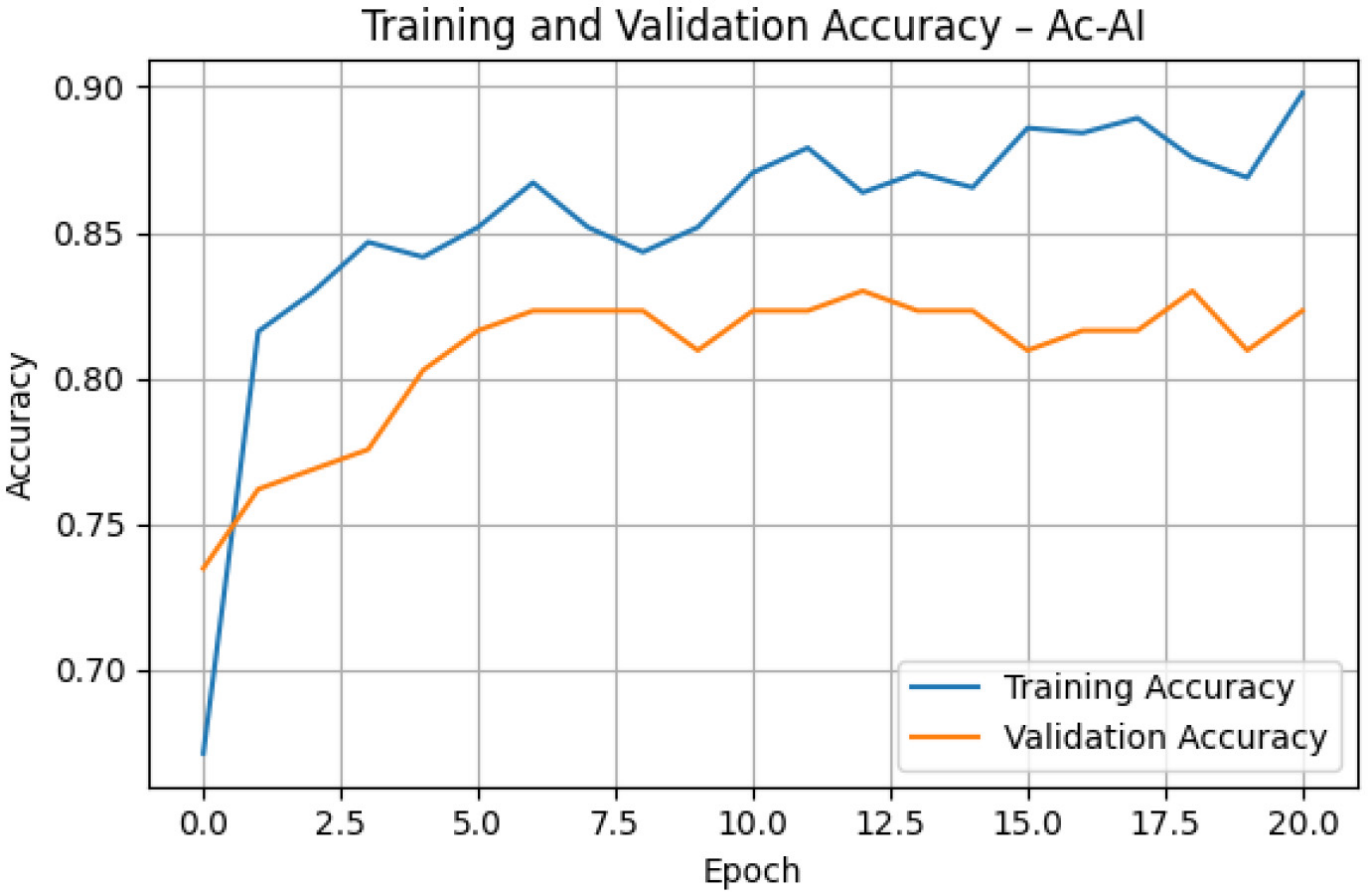
Training and validation accuracy.

**Figure 7:**
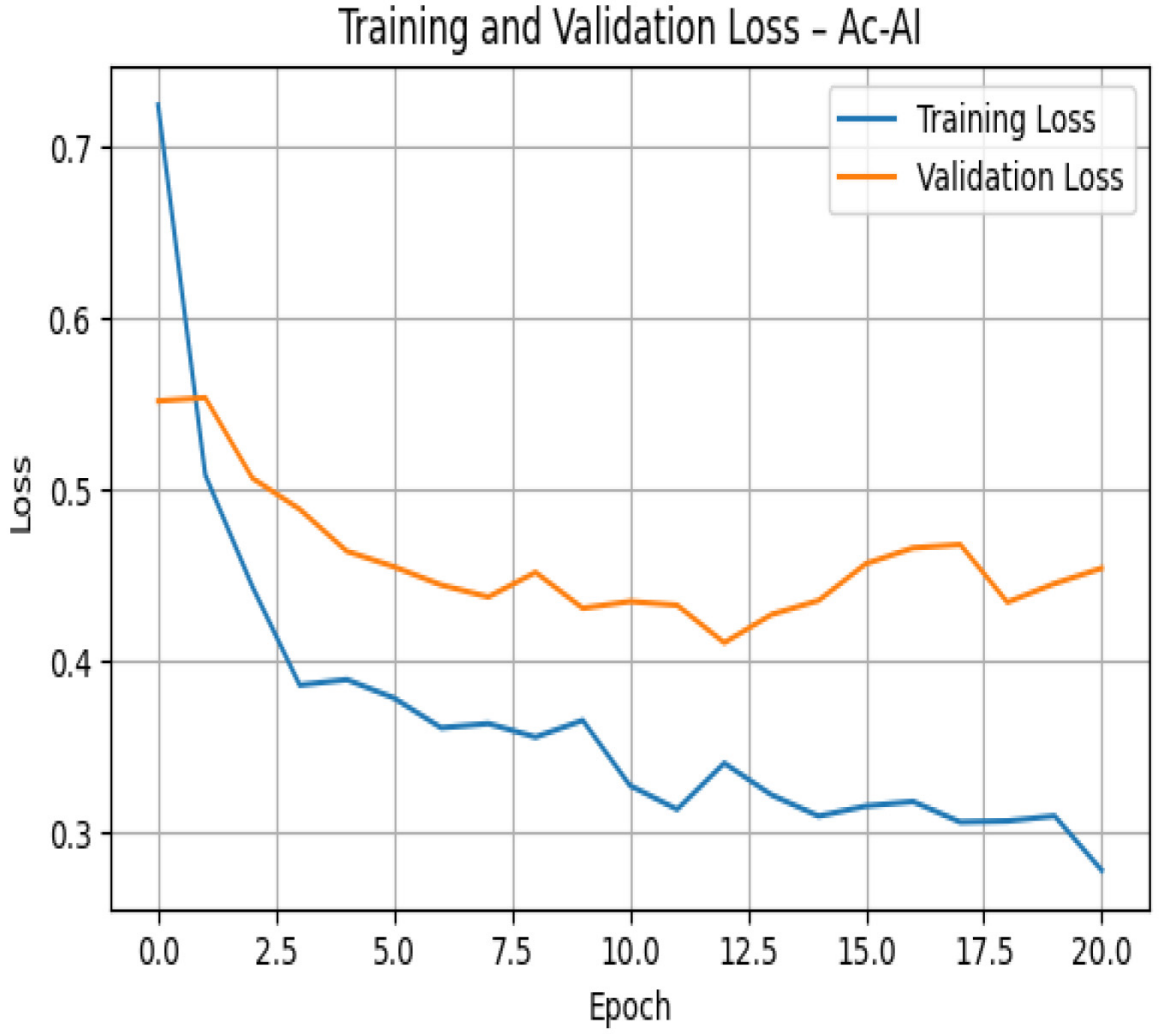
Training and validation loss.

Training stability and convergence behavior were evaluated using learning curves that track the training and validation AUC, along with loss values across epochs, as shown in Figures 6, 7. The curves exhibit smooth convergence trends without sudden fluctuations, suggesting stable optimization behavior.

Close agreement between training and validation performance indicates that overfitting was well controlled through regularization methods such as dropout and early stopping. These results confirm that the deep feature engineering approach improves model ability without compromising generalization. Similar patterns were seen in our experiments across different data splits.

### 5.6 Comparative performance visualization

**Figure 8:**
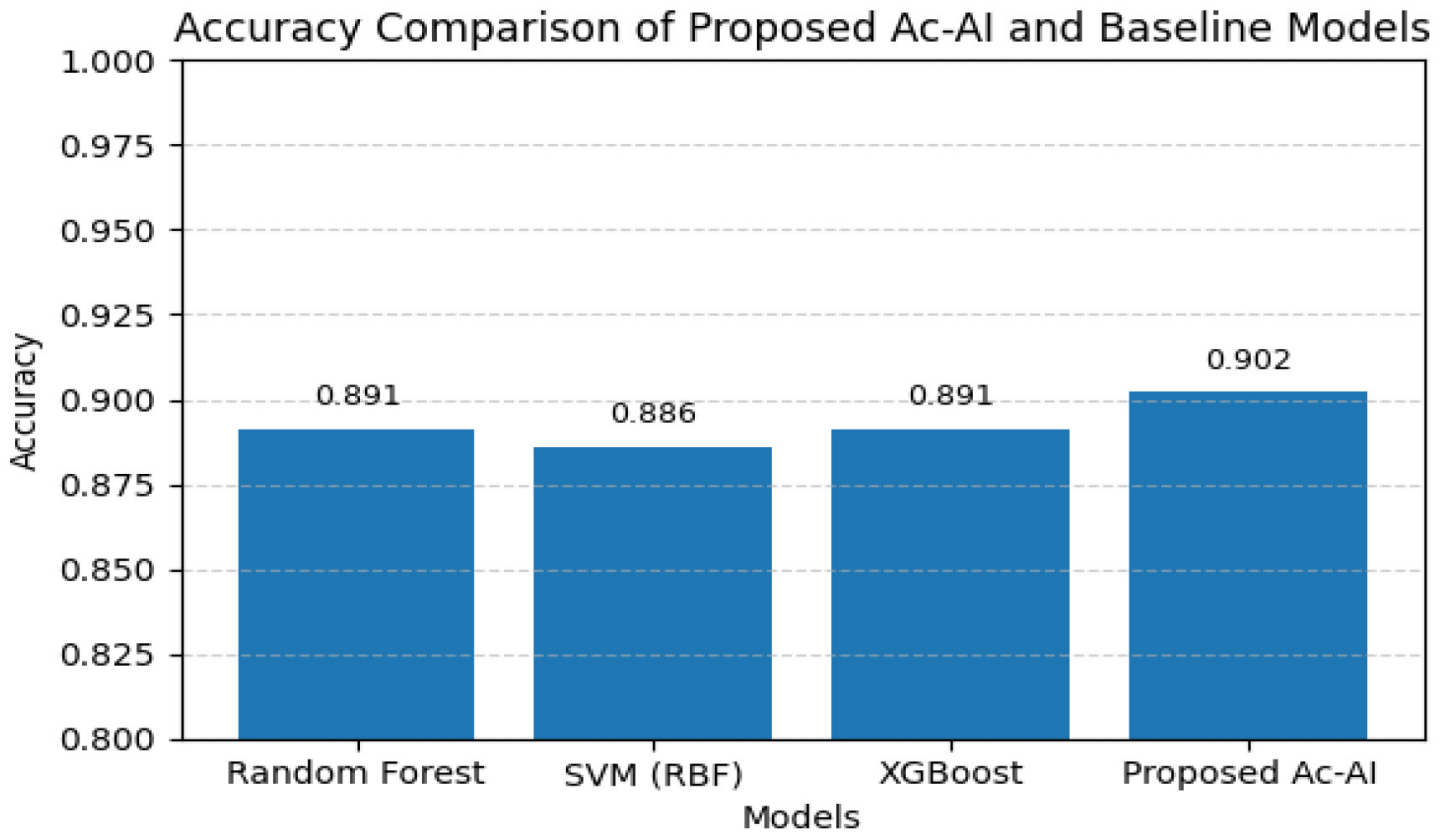
Accuracy comparison bar chart of all models.

**Figure 9:**
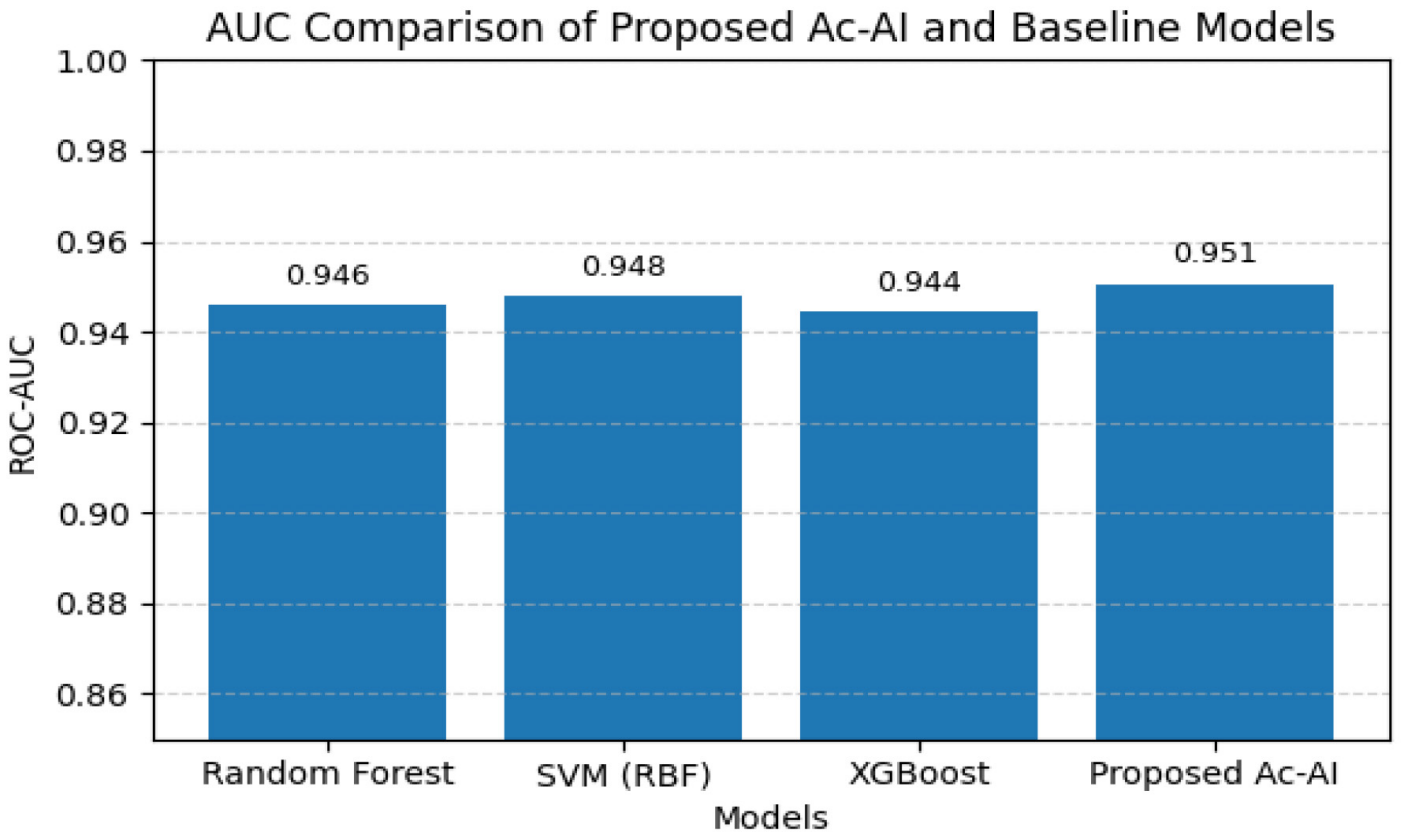
AUC comparison bar chart of all models.

To provide a clear comparison among models, Figure 8 presents a bar chart visualization of classification accuracy, while Figure 9 compares AUC values. These visual summaries reinforce the numerical results reported earlier and show that the proposed Ac-AI framework performed consistently, and in some cases slightly better than the selected baseline models.

Such visual comparisons support the claim that integrating acentric decision intelligence and adaptive learning mechanisms improves consistency without introducing instability into the prediction process.

### 5.7 Ablation study

To determine the individual contributions made by each module in the Ac-AI framework, an ablation analysis was performed by progressively adding the deep autoencoder-based feature learning component, cost-sensitive optimization, and adaptive threshold adjustment to the proposed model.

The ablation study results shown in Table 3 demonstrate the contribution of the individual components of the proposed Ac-AI framework. The baseline neural classifier achieved an accuracy of 0.8814, recall of 0.9032, F1-score of 0.8921, and AUC of 0.9394. The system achieved better results after adding deep autoencoder-based feature extraction because hierarchical latent feature learning successfully captured clinically relevant heart disease dataset information. The incorporation of cost-sensitive learning improved disease-class detection by increasing recall to 0.9321, while F1-score and AUC also improved due to a reduction in false-negative predictions. The adaptive thresholding method provided two benefits by enhancing predictive performance and improving disease-based detection methods, increasing recall to 0.9378 and AUC to 0.9492. The complete Ac-AI framework achieved the best overall performance, with an accuracy of 0.9021, recall of 0.9411, F1-score of 0.9143, and AUC of 0.9513. The progressive improvements observed across all configurations confirm that each module contributes positively to the overall effectiveness of the proposed framework, while the combined integration of deep feature engineering, cost-sensitive optimization, and adaptive thresholding enables robust and clinically sensitive heart disease prediction.

**Table 3:** Ablation study of deep feature engineering, cost-sensitive learning, and adaptive threshold optimization within the proposed Ac-AI framework.

Configuration	Accuracy	Recall	F1-score	AUC
Baseline neural classifier	0.8814	0.9032	0.8921	0.9394
+Deep autoencoder features	0.8896	0.9187	0.9018	0.9448
+Cost-sensitive learning	0.8942	0.9321	0.9074	0.9471
+Adaptive thresholding	0.8983	0.9378	0.9112	0.9492
Full Ac-AI framework	0.9021	0.9411	0.9143	0.9513

### 5.8 Statistical robustness analysis

To reduce the impact of random variation and assess reliability, all models were evaluated across multiple independent runs. The mean and standard deviation of key performance metrics are reported in Table 4.

**Table 4:** Mean ± standard deviation of performance metrics over multiple runs.

Model	Accuracy	Recall	F1-score (disease)	AUC (disease)
SVM	0.8859 ± 0.006	0.8922 ± 0.008	0.8966 ± 0.004	0.9482 ± 0.003
RF	0.8913 ± 0.008	0.9208 ± 0.010	0.9075 ± 0.006	0.9461 ± 0.004
XGB	0.8913 ± 0.009	0.9216 ± 0.011	0.9038 ± 0.007	0.9444 ± 0.005
Ac-AI	0.9021 ± 0.007	0.9411 ± 0.009	0.9143 ± 0.005	0.9513 ± 0.003

The proposed Ac-AI framework showed relatively low variance across runs, indicating consistent generalization behavior. This stability can be attributed to the combination of deep feature representation learning and adaptive decision strategies, which together reduce sensitivity to data partitioning and initialization effects.

A paired *t*-test was performed to test if the observed improvements in performance were statistically significant using scores obtained during *k*-fold-wise validation. Statistical comparisons were carried out using accuracy, recall, F1-score, and ROC–AUC, and the significance level was set at a *p*-value of < 0.05. The statistical analysis demonstrated that the proposed Ac-AI framework achieved statistically significant improvements over SVM, RF, and XGBoost across multiple evaluation metrics. The proposed adaptive cost-sensitive decision learning mechanism for heart disease prediction tasks proved effective because SVM testing showed a highly significant disease-class recall improvement. The detailed statistical comparison results are presented in Table 5.

**Table 5:** Statistical significance analysis.

Comparison	Metric	*t*-value	*P*-value	Significance
Ac-AI vs. SVM	Accuracy	3.284	0.01	Significant
Ac-AI vs. SVM	Recall	5.912	< 0.001	Highly significant
Ac-AI vs. SVM	F1-score	3.674	0.006	Significant
Ac-AI vs. SVM	AUC	2.847	0.019	Significant
Ac-AI vs. RF	Accuracy	2.194	0.056	Marginal
Ac-AI vs. RF	Recall	2.508	0.034	Significant
Ac-AI vs. RF	F1-score	1.768	0.111	Not significant
Ac-AI vs. RF	AUC	2.114	0.063	Marginal
Ac-AI vs. XGB	Accuracy	2.816	0.02	Significant
Ac-AI vs. XGB	Recall	3.447	0.008	Significant
Ac-AI vs. XGB	F1-score	2.605	0.028	Significant
Ac-AI vs. XGB	AUC	3.006	0.015	Significant

Statistical significance was assessed using paired two-tailed *t*-tests based on repeated experimental runs, meaning we compared the results more than once. A cutoff of *p* < 0.05 was used to decide if the improvements we saw were actually statistically meaningful, and not just a fluke. Evaluation across repeated runs reduces sensitivity to random data partitioning and model initialization effects, thereby improving the overall reliability of the comparative analysis.

### 5.9 Cross-dataset generalization

Table 6 presents the cross-dataset generalization performance of the proposed Ac-AI framework and baseline models on the multi-center heart disease dataset, comprising clinical records from Cleveland, Hungary, Switzerland, and Long Beach VA. The evaluation shows that the proposed Ac-AI framework exhibited stable and robust predictive performance when tested across diverse clinical data distributions from multiple centers. Among all tested models, the proposed Ac-AI framework achieved the best overall performance, with overall accuracy (0.9136), precision (0.9141), and F1-score (0.9152), indicating higher classification consistency and predictive capability. For instance, XGBoost achieved a slightly higher recall (0.9263) compared to some traditional models; however, the proposed Ac-AI framework also demonstrated strong recall performance (0.9320), along with superior overall classification stability and feature representation capability. In addition, the proposed framework attained an AUC value of 0.9555, suggesting excellent discriminative performance across different clinical subsets. The consistency in performance across independent multi-center datasets indicates that the proposed framework generalizes beyond a single benchmark dataset and has the potential to be used in clinical heart disease risk assessment applications.

**Table 6:** Cross-dataset performance evaluation.

Model	Accuracy	Precision	Recall	F1-score	AUC
SVM	0.8849	0.9043	0.8948	0.8996	0.9582
Random Forest	0.8953	0.8694	0.9246	0.9075	0.9571
XGBoost	0.8933	0.8779	0.9263	0.9088	0.9524
**Ac-AI (proposed)**	**0.9136**	**0.9141**	**0.932**	**0.9152**	**0.9555**

Bold values represent the best-performing results for each evaluation metric.

### 5.10 SHAP-based explainability analysis

**Figure 10:**
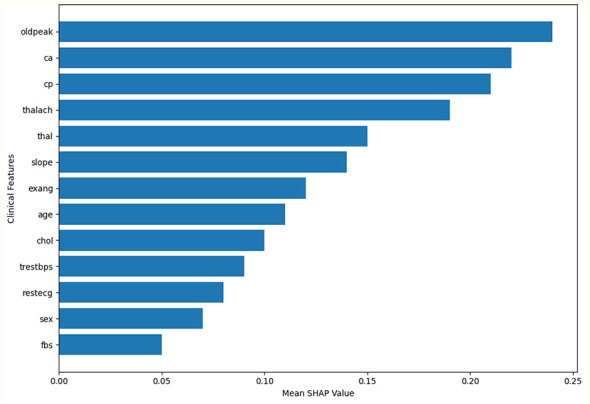
SHAP feature importance analysis of the proposed Ac-AI framework.

Figure 10 shows the global SHAP feature importance analysis of the developed Ac-AI model. It depicts the mean importance of clinical features in relation to the prediction results for heart diseases. Features such as oldpeak, number of major blood vessels (ca), type of chest pain (cp), maximum heart rate achieved (thalach), and thal were found to be the key variables contributing to the predictions made by the model. Higher SHAP values indicate a greater influence on the prediction process. The findings suggest that the proposed Ac-AI model accurately identifies clinically significant cardiovascular risk factors with consistent prediction accuracy. The prominence of clinically relevant variables indicates that the adaptive cost-sensitive learning approach efficiently focuses on clinically important patterns related to heart disease risk.

**Figure 11:**
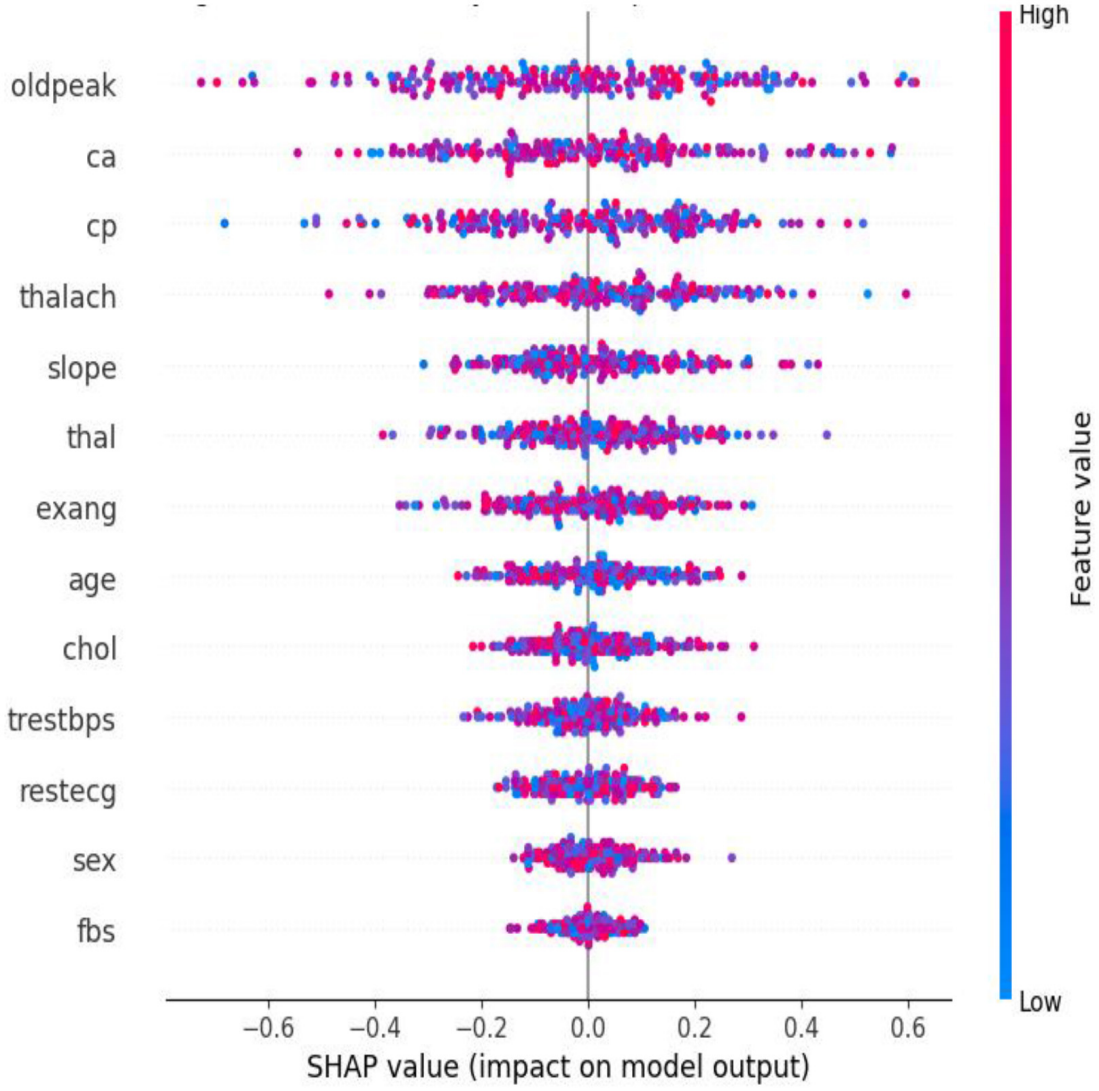
SHAP summary plot of the proposed Ac-AI framework.

Figure 11 shows the SHAP summary plot, which provides a comprehensive visualization of feature influence across all test samples. Each point represents the SHAP contribution of a feature for an individual patient, while the color gradient indicates the relative feature value from low to high. Positive SHAP values increase the probability of heart disease prediction, whereas negative values reduce the predicted risk. The summary plot indicates that higher values of oldpeak, ca, and exang contribute strongly to positive disease predictions, while increased thalach values are generally associated with decreased predicted risk. The distribution of SHAP values across samples demonstrates that the proposed Ac-AI framework captures non-linear and patient-specific decision behavior rather than relying on rigid centralized decision boundaries. This characteristic supports the framework's suitability for clinically adaptive risk prediction applications.

### 5.11 Comparison with existing studies

The comparison between the proposed Ac-AI framework and other heart disease prediction models reported in the literature is presented in Table 7. The Gaussian process classifier (GPC) had an accuracy rate of 89.91%, whereas the SAE-CNN model exhibited an accuracy rate of 90.09%. In contrast, the proposed Ac-AI framework showed the best accuracy rate of 90.21%. This suggests that the proposed heart disease prediction model outperforms other models by employing deep latent feature extraction, cost-sensitive optimization, and adaptive thresholding.

**Table 7:** Performance comparison with existing studies.

Model	Accuracy	References
Gaussian process classifier (GPC)	89.91	Chanrasekaran et al., 2025
SAE-CNN	90.09	Benavides et al., 2023
**Ac-AI (proposed)**	**90.21**	**This study**

Bold values represent the best-performing results for each evaluation metric.

### 5.12 Discussion

The empirical results indicate that the proposed Ac-AI framework is effective in delivering stable and clinically meaningful performance for early-stage heart disease risk prediction. The Ac-AI classifier demonstrated superior performance in disease-class recall, F1-score, and ROC-AUC measures compared to traditional machine learning models such as support vector machines (SVM), random forest (RF), and extreme gradient boosting (XGBoost). Despite the relatively small numerical gains in overall accuracy and area under the curve (AUC) metrics, the consistent improvement in recall is particularly significant in clinical screening tasks, where reducing false negatives is more crucial than achieving maximum overall accuracy. This is mainly because the Ac-AI framework incorporates feature engineering using an autoencoder, cost-sensitive learning, and adaptive thresholding. The use of an autoencoder improves the quality of feature representation by discovering non-linear correlations between clinical features while preserving clinical semantics through feature fusion. Meanwhile, cost-sensitive learning assigns higher weights to disease-class samples, which enables greater sensitivity to high-risk patients, whereas adaptive thresholding optimizes decision behavior beyond traditional threshold-based classification. The ablation analysis further confirmed that each component contributed positively to performance improvement, with the full Ac-AI framework achieving the best overall results. Statistical robustness analysis showed low variance across repeated runs, and paired significance testing demonstrated that several improvements, particularly in recall performance, were statistically significant compared to baseline models. Furthermore, cross-dataset validation experiments demonstrated stable predictive capability across independent multi-center heart disease datasets, suggesting that the framework possesses reasonable generalization ability beyond a single benchmark dataset. Despite these promising outcomes, certain limitations remain, including reliance on publicly available datasets with moderate sample sizes, relatively small performance margins over strong ensemble baselines, and the use of a comparatively shallow autoencoder architecture. Therefore, future research should focus on validation using larger real-world clinical datasets, incorporation of explainable AI methods such as SHAP analysis for interpretability, and exploration of more advanced deep representation learning strategies to further enhance robustness and clinical applicability. We conducted repeated experimental runs and statistical significance testing to improve the reliability of the evaluation. However, stronger statistical generalization requires validation on larger real-world clinical datasets and multi-center validation studies.

## 6 Conclusion

In this study, we present an early heart disease risk prediction framework based on acentric artificial intelligence (Ac-AI) integrated with deep feature engineering. The proposed method employs autoencoder-based feature learning to derive compact latent features from clinical data, which are fused with the original attributes to improve feature expressiveness. By incorporating cost-sensitive learning and adaptive decision threshold optimization, the Ac-AI classifier enhances disease-class sensitivity, addressing a key requirement in clinical decision support applications.

Comprehensive experimental evaluation on a benchmark heart disease dataset showed that the proposed framework delivers stable and competitive performance compared to widely used baseline models, including RF, SVM, and XGBoost. Visual evaluations using ROC curves, precision–recall curves, confusion matrices, and convergence plots further validated the robustness of the model and its ability to reduce missed disease cases. Statistical validation across multiple independent runs indicated consistent generalization behavior with limited performance variation.

Overall, the findings suggest that integrating deep feature engineering with acentric decision learning provides a reliable and scalable solution for early heart disease risk prediction. Unlike conventional centralized classifiers, the proposed framework adapts its decision behavior based on data distribution and misclassification costs. It is particularly suitable for medical prediction tasks where early risk sensitivity is critical.

## 7 Future research

Future research will extend this framework toward dynamic and time-evolving healthcare environments. In particular, integrating the proposed Ac-AI model with digital twin-based patient models may support continuous health monitoring and personalized risk assessment. Future studies will investigate advanced data augmentation strategies, including generative models coupled with intelligent optimization techniques, to improve model stability and generalization. Validation on larger multi-center clinical datasets and real-world electronic health records will also be pursued to assess scalability and clinical applicability.

## Data Availability

Publicly available datasets were analyzed in this study. This data can be found at: http://archive.ics.uci.edu/ml.
